# Reassessing the Risks of Tamiflu Use during a Pandemic to the Lower Colorado River

**DOI:** 10.1289/ehp.11407

**Published:** 2008-07

**Authors:** Andrew C. Singer, Andrew C. Johnson, Paul D. Anderson, Shane A. Snyder

**Affiliations:** Centre for Ecology & Hydrology, Oxford, United Kingdom, E-mail: acsi@ceh.ac.uk; Centre for Ecology & Hydrology, Wallingford, United Kingdom; AMEC Earth and Environmental, Westford, Massachusetts; Southern Nevada Water Authority, Henderson, Nevada

We wish to highlight an error in the article “Potential Risks Associated with the Proposed Widespread Use of Tamiflu” (Singer et al. 2007) in which we predicted environmental concentrations of Tamiflu (influenza antiviral) in several catchments in the United States and the United Kingdom. An incorrect assumption was made in the hydrology of one of these catchments, the Lower Colorado River (LC).

In that study (Singer et al. 2007), we used flow data generated by the U.S. Geological Survey (USGS) and presented in BASINS [U.S. Environmental Protection Agency (EPA) 2001], as well as watershed population statistics summarized by Anderson et al. (2004). We (Singer et al. 2007) presented the flow of the LC as 1,223,424 m^3^/day, serving a population of 5,861,200, thereby providing 0.2 m^3^/person/day of available dilution. These values were used to determine the predicted environmental concentration of oseltamivir carboxylate (OC), the active antiviral metabolite of the prodrug oseltamivir phosphate (Tamiflu), in the river during an influenza pandemic.

Unlike the other rivers investigated in the study, the mouth of the LC is in Mexico. Owing to its legal requirement as per Article 15 of the U.S.–Mexican Water Treaty (U.S. Government 1944), the United States releases 1,850,234,000 m^3^/year of the Colorado River to Mexico (Matuska 2007; U.S. Government 1944). This flow equates to 5,069,134 m^3^/day, which is roughly 5-fold higher than the values used in our previous study (Singer et al. 2007). Hence, the predicted environmental concentration of OC in the LC was considerably overestimated.

Detailed characterization of pollution risks in the LC is particularly challenging because of *a*) the arid environment and high evaporation; *b*) water conservation efforts lending many rivers to run dry; and *c*) numerous diversions for irrigation and domestic use. A survey of the rivers that join the LC indicate few, if any, significant inflows into the LC from major metropolitan areas downstream of Las Vegas (USGS 2008). Notably, the only other major city that might feed into the LC is Phoenix, which discharges into the Gila River. The daily mean discharge of the Gila River where it joins the LC is 464.8 m^3^/day (in 2007), thereby augmenting the LC flow by < 10% (USGS 2008). Hence, risk characterization of this catchment was focused on the sewage discharge from Las Vegas to the LC.

Las Vegas lies within Clark County, Nevada, and has a population of 1,996,542 (Clark County Department of Comprehensive Planning 2007). Wastewater treatment plants from Clark County produce approximately 757,000 m^3^/day, which is consistent with a population of 2,102,777, assuming 360 L/person/day, as is consistent for U.S. water usage patterns [Water Services Regulation Authority (OFWAT) 2007]. The wastewater is discharged into the Las Vegas Wash, a reach of Boulder Basin containing Lake Mead; Lake Mead has a storage capability of 17,500,000,000 m^3^ of water (Matuska 2007).

In the event of an influenza pandemic with a clinical infection rate of 35%, and assuming 100% pharmaceutical coverage of the infected population, approximately 3.0 ng OC would accumulate in each liter of water in Lake Mead. Expectations are such that only 25% of the infected population will receive Tamiflu (U.S. Department of Health and Human Services 2006), resulting in < 1 ng/L in Lake Mead. Given the average 3.9-year retention time within Lake Mead (LaBounty and Burns 2005; LaBounty and Horn 1997), the OC concentrations will accumulate (OC is poorly biodegradable) in the lake over the course of a pandemic—unlike in rivers, which were previously modelled (Singer et al. 2007). Hence, risk characterization of the LC indicates that the predicted environmental concentration of OC will be well below levels known to induce viral resistance (Aoki et al. 2007; Hurt et al. 2007).

One remaining concern is the poor mixing and highly stratified nature of Lake Mead; for example, the wastewater input into the lake is often slow to mix completely with the rest of the water column (LaBounty and Horn 1997), resulting in 40-times greater perchlorate levels in the themocline (30–40 m depth), than in the epilimnion or hypolimnion (LaBounty and Burns 2005). Such observations can be used to predict that OC concentrations > 80 ng/L may be expected in the thermocline in a pandemic situation. However, because wastewater comprises only 1.5% of Lake Mead’s flow and because the water in Lake Mead is discharged through the Hoover Dam from the hypolimnion, the location of lowest pollution, there is a low risk to the environment as a consequence of OC release.

Although we are pleased that the risk to the LC is lower than originally reported, this reinterpretation does not change the overall thrust of the article (Singer et al. 2007): After an influenza pandemic, OC concentrations in rivers would be considerably greater than previously seen for any other pharmaceutical, with the actual impact varying based on dilution within a catchment.
